# Preventable multiple high-risk birth behaviour and infant survival in Nigeria

**DOI:** 10.1186/s12884-021-03792-8

**Published:** 2021-05-01

**Authors:** Mobolaji Modinat Salawu, Rotimi Felix Afolabi, Babatunde Makinde Gbadebo, Adetokunbo Taophic Salawu, Adeniyi Francis Fagbamigbe, Ayo Stephen Adebowale

**Affiliations:** Department of Epidemiology and Medical Statistics, Faculty of Public Health, College of Medicine, University of Ibadan, Ibadan, Nigeria

**Keywords:** Infant mortality, High risk birth, Child health, Nigeria

## Abstract

**Background:**

Globally, infant mortality has declined considerably but has remained unacceptably high in sub-Saharan Africa, especially Nigeria where infant mortality rate is 67/1000 live births. To facilitate infant mortality reduction in Nigeria, an understanding of the synergistic effect of bio-demographic characteristics of mothers known as High Risk Birth Behaviours (HrBBs) is important. We therefore investigated the influence of HrBBs on infant survival in Nigeria.

**Methods:**

This cross-sectional study design utilized data from the 2018 round of Nigerian Demographic Health Survey. The study participants were a representative sample of women of reproductive age (*n* = 21,350) who had given birth within the 5 years preceding the survey. HrBBs was measured through integration of information on maternal age at child’s birth, parity, and preceding birth interval with respect to the most recent child. The HrBBs was categorized as none, single and multiple. Data were analysed using descriptive statistics, Log-rank test and Cox proportional hazard model (α =0.05).

**Results:**

The mean age of the women was 29.7 ± 7.2 and 4.1% had experienced infant death. Infant mortality was highest among women with multiple HrBBs (5.1%). Being a male, having small size at birth, failure to receive tetanus injection, non-use of contraceptives and living in the core-north (North West and North East) predisposed children to higher risk of dying before 12 months of age. The hazard ratio of infant mortality was significantly higher among infants of mothers in multiple HrBBs category (aHR = 1.66; CI: 1.33–2.06) compared to their counterparts with no HrBBs.

**Conclusion:**

Multiple HrBBs increase the chances of dying among infants in Nigeria. Screening women for HrBBs for special health attention during pregnancy, birth and postnatal period will alleviate infant death in Nigeria.

## Introduction

Infant Mortality which is the probability of a child dying between birth and the first birthday is one of the most useful indicators for assessing the general level of health and development of a society. It gives an overview of the functionality of a country’s healthcare system, socioeconomic situation, and the state of maternal and child health. Globally, 85 and 29% of deaths among children occurred in the first 5 years of life and during infancy respectively. More than 50 % of these deaths occurred in Sub-Sahara Africa (SSA) with Infant Mortality Rate (IMR) of 62 deaths per 1000 in 2018 [1]. Improvement in child health, survival and life expectancy has been a concerted and continuous global effort as indicated in the Sustainable Development Goals – 3 (SDGs 3) which aims to end preventable deaths among children under-5 years of age and targets reduction of under-five mortality to as low as 25 per 1000 live births by 2030 [2]. However, feasibility of the realization of this target is doubtful due to slow pace of mortality reduction in SSA as many countries in this world sub-region may likely fall short of the SDG target [1].

Nigeria with a population of over 200 million is one of the five countries that accounted for half of global burden of infant mortality occupying a second position after India [1, 3]. In Nigeria, previous studies have estimated a decline in IMR from 125 in 1990 to 67 in 2018 [3, 4]. Despite this achievement in IMR reduction over the years, the current level is higher than the IMR estimates for other countries in SSA like South Africa (28/1000), Kenya (31/1000) and Ghana (35/1000) which are already close to achieving the SDGs - target 3. Survival of infants in Nigeria is challenged by the prevailing poor health service delivery and malnutrition as a result of poverty which ravages the nation. Some preventable health/environmental related conditions (infectious diseases, chronic health conditions of the mother, obstetric and non-obstetric complications, lack of immunization, and other prevalent childhood diseases), socio-demographic characteristics (place of residence, region, religion, marital status and education level) and biological factors associated with mothers have been found in the literature as additional sources of threat to the survival chances of infants in Nigeria [1, 5–7]. It is important to note that risk of adverse pregnancy outcome like infant mortality are unevenly distributed among women population owing to the variation in their biologic features and demographic composition [8].

High-risk Birth Behaviours (HrBBs) in the context of this study was defined in terms of three preventable characteristics, namely; mother’s age at delivery which can either be too young (less than 18 years) or too old (greater than 35 years), shorter or longer birth interval, and a high parity [9, 10]. These characteristics have been a major public health challenge to child survival in developing countries particularly Nigeria where little or no efforts are put in place to improve the health status of children. Studies have reported that teenage pregnant mothers have a greater risk of having pre-term birth, low birth weight, stillbirths and more importantly infant mortality [8, 11–13]. These unacceptable birth outcomes and child health have been linked to biological vulnerability, socioeconomic factors or inexperience of mothers in terms of pregnancy and child care management such as exclusive breastfeeding and nutritional support [8]. Women’s age at marriage has somewhat increased in Nigeria with a resultant increase in age at childbirth owing to the impact of the socioeconomic changes, such as improvements in school enrolment, increased modern contraceptives use and economic opportunities [13]. Studies have reported that women who gave birth at ages 35 years and above have a higher risk of infant mortality [14–17]. A study by Yogev et al. to determine pregnancy outcome at advanced maternal age reported that advanced maternal age of greater than 35 years is associated with higher risk of maternal, hormonal disorder, and low uteroplacental blood flow which increases the risk of congenital and chromosomal abnormalities such as downs syndrome that results in fetal complications with resultant infant death [17]. Studies showed that children born to young mothers (under age 18), had a 20% greater risk of dying in the first year of life, and children born to mothers ages 35–39 and 40 or older have a 20% and 50% greater risk of dying in the first year of life, respectively [9, 18, 19].

The established relationship between infant mortality and birth interval has been consistent in the literature. Short and longer birth interval increased the risk of infant mortality, often referred to as a U-shape hypothesis [9, 20, 21] Inability of the mother to give adequate care to the index child in terms of nutrition, optimal health and support, especially if the preceding child was born not too long ago has been found to be responsible for the direction of the association [9]. In a poverty-stricken environment like Nigeria, children born to high parity women may not have access to adequate care and this could impact negatively on their survival chances [10, 22]. The Nigerian population policy emphasizes that family size should be restricted to not more than four children [23]. Unfortunately, studies conducted in Nigeria after the enactment and implementation of this policy reported that most families still have more than the stipulated family size with a total fertility rate of 5.3 [24]. In a high family size situation, the harsh socioeconomic condition in Nigeria could limit an individual’s ability to provide basic needs for the family, particularly for infants who are the most vulnerable to morbidity and mortality [7, 13]. Moreover, some women exhibit more than one of these HrBBs at the same time which increases their risk of having children that are prone to death before the age of 1 year [11, 24, 25].

Studies have been conducted in Nigeria based on the independent analysis of maternal age at childbirth, short birth interval, high parity and infant survival [18]. However, very few studies examined the relationship between the combination of these factors as an entity and infant mortality. Against the backdrop of limited research on the association of HrBBs and infant mortality, the current study investigated the influence of the HrBBs (mother’s age at childbirth < 18 years or > 34 years, preceding birth interval < 24 months or > 59 months, and number of children ever born > 4) on infant mortality. The specific objectives of the study are to; assess the association between HrBBs and infant mortality in Nigeria, to examine if HrBBs is a predictor of infant mortality amidst other factors. The outcome of this study will inform policy geared towards the improvement of survival chances among children in Nigeria.

## Methodology

### Study design, area and population

The present study utilized 2018 Nigeria Demographic and Health Survey (NDHS) dataset. This cross-sectional and nationally representative sample survey was designed to provide information on population, maternal and child health indicators’ estimates, and fertility-related behaviours [3]. The study was conducted in Nigeria, African most populous country with about one in every 15 children dies before their first birthday [3, 26]. Nigeria has one of the highest Total Fertility Rates (TFR) worldwide (TFR of over 5 children per woman unabated) and less than 15% of women using modern contraceptive method. The birth intervals decline from 33.4 months in 2008 to 30.9 months in 2018 while the percentage of adolescent childbearing has slowly declined over the last decade (23% in 2008 vs 19% in 2018) in Nigeria [3, 27]. Presently, 80% of women of reproductive age engaged in a high-risk birth behaviours [3].

The two-stage cluster sampling technique was used for the survey based on the sampling frame adopted from the 2006 Nigeria population and housing census. The primary sampling units, referred to as clusters, were the enumeration areas. In all, 42,000 households were sampled from 1400 clusters selected for the survey. A detailed report of the sample design and sampling procedures has earlier been published [3]. The study analysis focused on the birth history of 33,924 women who had a recent birth within the 5 years preceding the 2018 NDHS. Women who did not respond to questions that are related to outcome and the main independent variables were excluded from the analysis. Analyses were restricted to a single-birth lastborn infant, and as such the study population consisted of 21,350 children aged 0–11 months who had valid and complete information on HrBBs variables (mothers age at childbirth, preceding birth interval and the total number of children ever born), date of birth and date of death.

### Study variables

The outcome variable of interest is the infant survival status (alive =0; death = 1). The survival time (months) was derived for each child aged 0–11 months using survival status at 12th month, age at death, and date of interview. Children who did not experience death as at the time of the survey were right-censored and were coded 0; otherwise, 1 in the analysis. The main explanatory variable was High-risk Birth Behaviour (HrBBs) derived from mother age at childbirth < 18 years or > 34 years, preceding birth interval < 24 months or > 59 months, and number of children ever born > 4. An indicator variable was generated for each of the three risky behaviours: mother age at childbirth was coded as 1 if age < 18 years or > 34 years, 0 otherwise; birth interval coded as 1 if interval < 24 months or > 59 months, 0 otherwise; children ever born as 1 if parity ≥5 and 0 if otherwise. The sum of the indicator variables was 3, 2, 1 or 0; ≥2 was categorised as multiple, 1 as single and 0 as non-high-risk birth behaviours.

Other covariates that were considered important according to literature and were included in the analyses include: household/environmental characteristics (regions, residence, ethnicity, religion, wealth quintile, type of cooking fuel, source of drinking water and type of toilet facility), child/mother characteristics (child’s sex, birth size, mother’s education, employment, marital status, desire for child), and health service-related characteristics (contraceptive use, decision-making involvement, number of ANC visits, tetanus injection received, breastfeeding initiation, health facility perception, place of delivery, prenatal attendant, type of delivery, and birth attendant) [25].

### Statistical methods of analysis

Descriptive statistics and survival analysis were used for the analysis. Data were weighted before use due to the complex sampling design used during data collection in order to adjust for differences in population sizes of each region in Nigeria. Weighting the data will extrapolate the analysis output to other areas not covered during the survey in order to enhance generalizability of the study’s findings. At univariate level, descriptive statistics were used to describe infant mortality by the key variable (high-risk birth behaviours status) and other selected covariates. The log-rank test was used to examine the relationship between survival status and the selected characteristics. The crude Cox PH model was used to explain the relationship between infant mortality and high-risk birth behaviours. **Cox proportional hazards (CPH) model:** The CPH modelled the hazard function as the dependent variable to determine which combination of explanatory variables significantly affects the hazard. We thus expressed the CPH model with the predictors as:

$$ h\left({t}_i\right)={h}_0(t){\ell}^{b_1{x}_{1i}+{b}_2{x}_{2i}+\cdots +{b}_p{x}_{pi}}={h}_0(t){\ell}^{\sum_{j=1}^p{b}_j{x}_{ji}} $$

$\overset{\kern1em imlying\kern1em }{\to}\mathit{\ln}\frac{h\left({t}_i\right)}{h_0(t)}={b}_1{x}_{1i}+{b}_2{x}_{2i}+\cdots +{b}_p{x}_{pi}={\sum}_{j=1}^p{b}_j{x}_{ji}$Where *b*_*j*_ - vector of the coefficients of the explanatory variables,

*h*_0_(*t*) - baseline hazard function and

$\frac{h(t)}{h_0\left(t\ \right)}$ - hazard ratio (HR).

The coefficients b_j_ indicates the changes in the expected time to infant death due to a unit change in the jth predictor. The exponentials of the coefficients suggest the tendency of exposure to infant death; thus, HR > 1 indicates higher exposure, HR < 1 lower exposure and HR = 1 equality.

Amidst other independent variables, four adjusted Cox PH models 1–4 were fitted. Models 1–3 encompassed variables to define household, child/maternal, and health service-related characteristics along with preceding model(s) significant variables, respectively; while model 4 included all the significant factors (*p* < 0.20) based on the log-rank test. The HRs and their respective 95% confidence intervals (CIs) were reported. In each of the models, Akaike Information Criteria (AIC) and Bayesian Information Criteria (BIC) values were also reported for model comparison; the model with the least value was adjudged as being more adequate [8].

Of note, prenatal attendant and place of delivery were excluded in the model due to collinearity with number of ANC visits and birth attendant variables, respectively. All analyses were carried out at 5% level of significance, using STATA 14 SE.

## Results

### Background characteristics of infants

Infant survival status by household, demographic and health service utilization characteristics are described in Table 1. Most infants (60.3%), lived in a rural area, 35.0% were from the North-west region and 46.6% were of Hausa/Fulani ethnicity. About half of the children (43.8%) were from poor wealth quintiles’ household; the majority (59.1%) resided in households with an improved source of drinking water and 89.8% had no improved cooking fuel. Half of the infants were male (51.2%) and 51.9% had reported average birth size. Nearly half and one-thirds of the infants had mothers who were uneducated (44.5%) and currently unemployed (31.7%), respectively. Almost all the infants had mothers who were ever married (97.6%). Majority of the infants received tetanus injection (69.9%), 15.0% delayed initiation of breastfeeding, 96.9% were delivered normally and 57.7% had mothers who visited ANC for at least 4 times. Most mothers (63.7%) received prenatal assistance from skilled personnel but 41.6% had their delivery attended to by unskilled personnel.

**Table 1:** Weighted percentage distribution of infant survival status by socio-demographic and health services utilisation characteristics

Characteristics	n (%)	Death (%)	***p***-value#	Characteristics	n (%)	Death (%)	*p*-value
**Household**				**Health service**			
**Region**			< 0.001*	**Contraceptive use**			< 0.001*
Northcentral	3791 (13.8)	3.8		Using	3520 (17.2)	1.9	
Northeast	4430 (17.7)	4.5		Not using	17,830 (82.8)	4.6	
Northwest	6198 (35.0)	4.7		**Decision involvement**^**+**^			0.064
Southeast	2301 (9.7)	3.4		None	8032 (39.3)	4.3	
Southsouth	2137 (9.2)	3.3		Low	10,050 (50.1)	3.9	
Southwest	2493 (14.6)	3.4		High	1913 (10.6)	3.6	
**Residence**			0.191	**ANC visit**^**+**^			< 0.001*
Urban	7543 (39.7)	4.0		None	5279 (24.9)	5.0	
Rural	13,807 (60.3)	4.1		1–3	3710 (17.4)	4.2	
**Religion**			< 0.001*	≥4	12,039 (57.7)	3.6	
Christianity	8721 (38.0)	3.4		**Tetanus injection**^**+**^			< 0.001*
Islam	12,456 (61.5)	4.5		Received	14,768 (69.9)	3.6	
Others	173 (0.5)	3.0		Not received	6478 (30.1)	5.4	
**Ethnicity**			0.009*	**Breastfeeding initiation**^**+**^			0.001*
Hausa/Fulani	9079 (46.6)	4.7		Not delayed	17,795 (85.1)	2.6	
Igbo	2761 (12.5)	3.5		Delayed	2969 (15.0)	3.6	
Yoruba	2285 (12.5)	3.3		**Health fac. perception**			0.337
Other	7225 (28.4)	3.7		Not-problem	9559 (46.6)	3.9	
**Wealth status**			< 0.001*	Problem	11,791 (53.4)	4.3	
Poorest	4947 (21.6)	4.4		**Place delivery**			0.042*
Poorer	4816 (22.2)	4.9		Hospital	9097 (42.7)	3.9	
Middle	4477 (20.2)	4.0		Home	12,253 (57.4)	4.3	
Richer	3939 (18.7)	3.8		**Prenatal attendant**			< 0.001*
Richest	3171 (17.2)	3.2		None	5279 (24.5)	5.0	
**Drinking water source**			0.018*	Unskilled	448 (2.5)	6.5	
Improved	12,273 (59.1)	3.8		Semi-skilled	2252 (9.4)	4.0	
Not improved	9077 (41.0)	4.5		Skilled	13,371 (63.7)	3.7	
**Type of toilet facility**			0.596	**Type of delivery**^**+**^			0.005*
Improved	10,424 (51.2)	4.0		Normal	20,650 (96.9)	4.0	
Not improved	10,926 (48.8)	4.2		Caesarean	610 (3.1)	6.1	
**Cooking fuel**^**+**^			0.205	**Delivery attendant**			0.038*
Improved	1742 (10.2)	4.0		None	2223 (10.8)	3.6	
Not improved	19,310 (89.8)	4.1		Unskilled	8995 (41.6)	4.4	
**Child/maternal**				Semi-skilled	1346 (6.1)	4.8	
**Sex**			0.003*	Skilled	8786 (41.6)	3.8	
Male	10,919 (51.2)	4.5					
Female	10,431 (48.8)	3.7					
**Birth size**^**+**^			< 0.001*				
Small	2818 (13.5)	5.5					
Average	10,923 (51.9)	3.8					
Large	7284 (34.5)	3.7					
**Highest education**			< 0.001*				
No education	9356 (44.5)	4.7					
Primary	3342 (15.1)	4.5					
Secondary/tertiary	8652 (40.4)	3.3					
**Employment**			0.122				
Working	14,488 (68.3)	4.1					
Not working	6862 (31.7)	4.2					
**Marital status**			0.441				
Not married/ in-union	600 (2.4)	5.0					
Married or in-union	20,750 (97.6)	4.1					
**Desire for last child**			0.165				
Then	18,670 (87.9)	4.2					
Later	1936 (8.8)	3.3					
No more	744 (3.3)	4.6					
*Total*	*21,350*	*4.1*		*Total*	*21,350*	*4.1*	

*significance at 5%; #based on log-rank test; ^+^missing value not reported

**Fig. 1:**
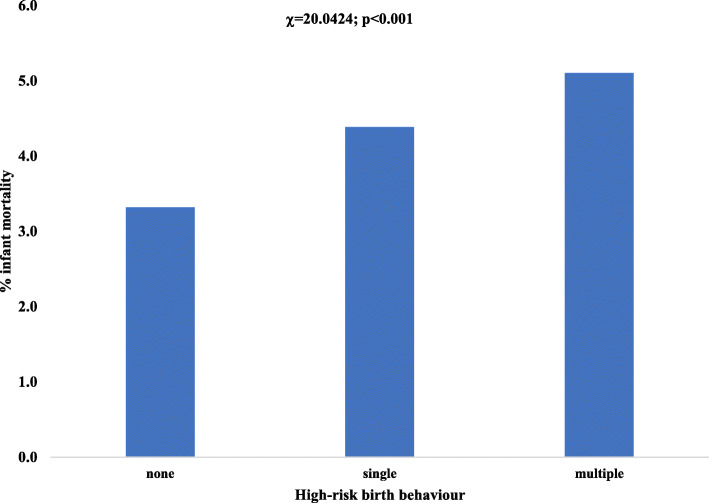
Infant death by categories of high-risk birth behaviour

In Fig. 1, the higher the number of HrBBs engaged by infants’ mothers the higher the percentage of infant mortality. Specifically, 5.1% of infant deaths occurred among mothers who engaged in multiple HrBBs compared with 4.4% infant death among mothers with single risk and 3.3% death among none high-risk birth behaviours. Of note, the association between infant death and HrBBs status (Wald chi-square statistic, χ = 20.0424; *p* < 0.001) was statistically significant.

**Fig. 2:**
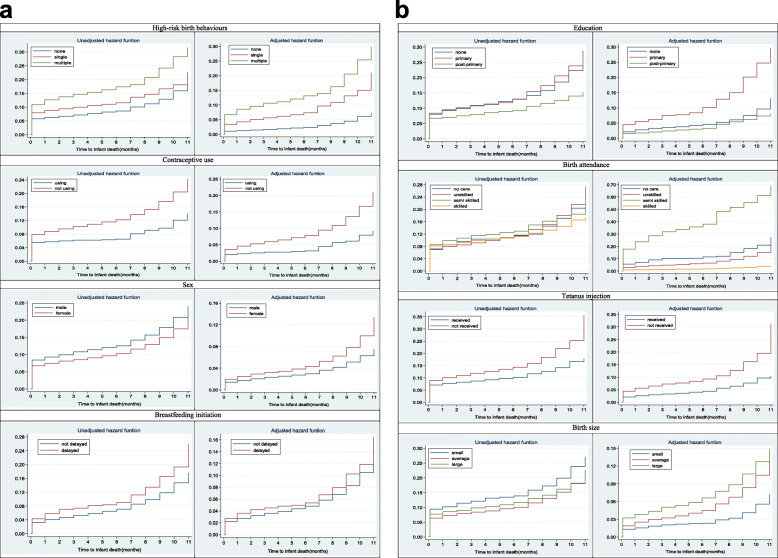
Hazard function of time to infant death

Figure 2 a and b show the cumulative hazard ratio by the high-risk birth behaviour and some selected variables.

### Association between background characteristics and infant mortality

Region of residence, religion, ethnicity, wealth status, source of drinking water, sex, birth size, maternal education, contraceptive use, ANC visit, tetanus injection, place and type of delivery, prenatal and delivery assistant, and breastfeeding initiation were significantly associated with infant mortality (*p* < 0.05). Specifically, the prevalence of infant death was common among mothers who did not use contraceptive (4.6%) relative to contraceptive users (1.9%). Children who were male (4.5%), of small size at birth (5.5%), born via caesarean (6.1%), and did not receive tetanus injection (5.4%) were more prone to infant death compared to their respective counterparts. Similarly, the prevalence of death was higher among infants whose mothers were uneducated (4.7%) and had zero visit to ANC (5.0%). Also, children from the North west (4.7%) and of Hausa/Fulani tribe (4.7%) were more vulnerable to die during infancy (Table 1).

### Influence of high-risk birth behaviours on infant survival status

The crude and adjusted models of time to infant death are shown in Table 2. The tendency of infant deaths increased as the number of HrBBs increases. Specifically, the hazards of infant mortality were significantly higher among mothers who engaged in single (HR = 1.23; CI: 1.05–1.44) and multiple (HR = 1.56; CI: 1.32–1.83) compared to those who engaged in no HrBBs as observed in the crude/simple model. The hazard of infant mortality remained significantly higher among infants whose mothers practiced multiple HrBBs while controlling for household, child/maternal and health service-related characteristics. Of note, the hazard of multiple HrBBs among mothers increased to 1.66 (aHR = 1.66; CI: 1.33–2.06) while selected health-related variables along with all significant variables at either model 1 or 2 were adjusted for (model 3 was preferred, having the lowest value of BIC = 10,130.7). Other significant predictors of infant death were, being a male, having small size at birth, non-recipient of tetanus injection, and whose mothers were non-contraceptive users, delayed breastfeeding initiation, or assisted by unskilled personnel at childbirth were. However, children whose mothers had at least secondary education level (aHR = 0.63; CI: 0.48–0.82) were less likely to die (as infants) compared to those whose mothers had no formal education.

**Table 2:** Factors associated with infant mortality

Characteristics	Unadjusted HR (95%CI)	aHR (95%CI)			
		Model1	Model2	Model3	Model4
**High risk behaviour**					
Non-risk	1	1	1	1	1
Single	1.23 (1.05,1.44)^a^	1.16 (0.99,1.36)	1.14 (0.96,1.34)	1.18 (0.96,1.47)	1.19 (0.96,1.48)
Multiple	1.56 (1.32,1.83) ^a^	1.46 (1.23,1.72) ^a^	1.43 (1.20,1.71) ^a^	1.66 (1.33,2.06) ^a^	1.66 (1.33,2.08) ^a^
**Region**					
Northcentral		1			1
Northeast		1.19 (0.94,1.51)			1.41 (1.03,1.92) ^c^
Northwest		1.22 (0.95,1.58)			1.44 (1.03,2.01) ^c^
Southeast		0.89 (0.53,1.52)			0.55 (0.24,1.26)
Southsouth		0.86 (0.63,1.17)			0.86 (0.54,1.37)
Southwest		0.92 (0.62,1.36)			0.69 (0.38,1.27)
**Residence**					
Urban		1			1
Rural		0.88 (0.74,1.04)			0.79 (0.62,1.00) ^c^
**Religion**					
Christianity		1			1
Islam		0.98 (0.78,1.23)			0.83 (0.60,1.15)
Others		0.80 (0.33,1.95)			0.58 (0.14,2.35)
**Ethnicity**					
Hausa/Fulani		1			1
Igbo		0.94 (0.56,1.59)			1.13 (0.51,2.51)
Yoruba		0.79 (0.51,1.21)			0.94 (0.49,1.78)
Other		0.94 (0.75,1.19)			0.91 (0.68,1.24)
**Wealth status**					
Poorest		1			1
Poorer		1.17 (0.96,1.41)			1.13 (0.90,1.42)
Middle		0.98 (0.78,1.23)			0.97 (0.73,1.28)
Richer		0.84 (0.64,1.12)			0.90 (0.63,1.29)
Richest		0.77 (0.54,1.09)			0.67 (0.42,1.08)
**Drinking water source**					
Improved		1			1
Not improved		1.10 (0.96,1.27)			1.13 (0.94,1.35)
**Toilet facility**					
Improved		1			
Not improved		0.92 (0.78,1.09)			
**Cooking fuel**					
Improved		1			
Not improved		0.79 (0.57,1.11)			
**Sex**					
Male			1.24 (1.08,1.42) ^b^	1.25 (1.05,1.49) ^c^	1.25 (1.05,1.49) ^c^
Female			1	1	1
**Birth size**					
Small			1.55 (1.29,1.85) ^a^	1.41 (1.11,1.79) ^b^	1.39 (1.09,1.77) ^b^
Average			1	1	1
Large			1.03 (0.89,1.20)	1.06 (0.87,1.28)	1.04 (0.86,1.26)
**Highest education**					
No education			1		1
Primary			0.95 (0.78,1.14)	1.07 (0.83,1.37)	1.23 (0.95,1.60)
Secondary/tertiary			0.67 (0.57,0.79) ^a^	0.63 (0.48,0.82)^a^	0.83 (0.60,1.13)
**Employment**					
Not working			1		1
Working			1.09 (0.94,1.26)		1.09 (0.90,1.32)
**Marital status**					
Not married/ in-union			1		
Married or in-union			0.67 (0.44,1.01)		
**Desire for last child**					
Then			1		1
Later			0.85 (0.65,1.12)		0.69 (0.44,1.07)
No more			0.99 (0.69,1.41)		1.20 (0.75,1.91)
**Contraceptive use**					
Using				1	1
Not using				1.86 (1.33,2.60) ^a^	1.79 (1.28,2.51) ^a^
**Decision involvement**					
Not				1	1
Low				0.94 (0.78,1.14)	1.99 (0.82,1.21)
High				1.04 (0.74,1.46)	1.15 (0.81,1.62)
**ANC visit**					
None				0.96 (0.69,1.33)	0.98 (0.70,1.37)
1–3				1.14 (0.89,1.46)	1.09 (0.85,1.39)
≥ 4				1	1
**Tetanus injection**					
Received				1	1
Not received				1.44 (1.08,1.93) ^c^	1.40 (1.04,1.88) ^c^
**Breastfeeding initiation**					
Not delayed				1	1
Delayed				1.27 (1.02,1.59) ^c^	1.25 (0.99,1.56)
**Type of delivery**					
Normal				1	1
Caesarean				0.99 (0.50,1.77)	1.05 (0.53,2.09)
**Delivery attendant**					
None				1	1
Unskilled				1.35 (1.02,1.77) ^c^	1.39 (1.05,1.83) ^c^
Semi-skilled				1.38 (0.89,2.15)	1.54 (0.98,2.42)
Skilled				1.20 (0.85,1.69)	1.45 (1.02,2.06) ^c^
-LL	8785.9	8671.6	8422.9	4976.8	4958.6
AIC	17,575.9	17,383.2	16,867.8	9989.6	9991.2
BIC	17,591.8	17,542.3	16,955.3	10,130.7	10,281.3
N	21,350	21,052	21,025	18,753	18,753

^c^ *p* < 0.05, ^b^ *p* < 0.01, ^a^ *p* < 0.001; place of delivery and pre-natal attendant removed due to collinearity

## Discussion

The global efforts with various intervention to reduce Infant Mortality Rate (IMR) has yielded progress in most parts of the world except in sub-Sahara Africa where infant mortality is still unacceptably high and account for more than 50 % of global infant death. Sadly, Nigeria is one of the five countries of the world that account for this poor statistic. Infant survival has been unduly affected by several preventable factors such as health/environmental related conditions, socio-demographic and biological characteristics of the mothers. Most importantly, the bio-demographic characteristics of mothers which is referred to as High-risk Birth Behaviours (HrBBs) has a strong impact on infant survival. This study therefore examined the influence of the combination of these three risk factors on infant survival in Nigeria.

Our findings showed that the higher the number of HrBBs engaged by infants’ mothers, the higher the likelihood of the infant mortality. Similar studies conducted in Nigeria substantiates this finding [13, 27]. A national survey in Bangladesh and multi-country studies also found that the more birth risks a child faces, the higher the risk of dying [9, 28]. Yogev et al. in a study to determine pregnancy outcome at advanced maternal age found out that advanced maternal age of greater than 35 years is associated with higher risk of maternal, hormonal disorder, and low uteroplacental blood flow which increases the risk of congenital and chromosomal abnormalities that results in fetal complications. In addition, children born to mothers aged 35–39 and 40 or older have a 20% and 50% greater risk of dying in the first year of life, respectively [29]. Studies showed that children born to young mothers (under age 18), had a 20% greater risk of dying in the first year of life [9, 17]. Young mothers have the tendency of having immature or underdeveloped physical and biological attributes with associated poor nutritional status which impact their reproductive capabilities. This has a direct effect on fetal development and survival.

Furthermore, the effect of a short birth interval of less than 24 months has been shown to affect infant survival, this harmful effects of a non–optimal preceding birth interval are concentrated in early infancy [20]. High parity also influences the chances of child survival as most mothers with many children tend to belong to lower socioeconomic status. They often have low or no formal education status, live in less favourable lifestyle, hence their children often suffer neglect [22]. The findings from previous studies on the association between each of the three factors used for the creation of HrBBs and infant survival was consistent with that of the combined HrBBs demonstrated in the current study [9, 25].

Additionally, other predictors of infant death found in our study were being a male child, mothers who did not receive tetanus injection, babies with small birth size, delayed breastfeeding initiation and unskilled birth attendant . This is consistent with research findings from similar studies [25, 30, 31]. An unskilled birth attendant is one of the health service delivery challenges in Nigeria. Unskilled birth attendants do not have the skill or expertise to risk stratify pregnancy, prevent or treat complications during childbirth or delivery. They are unable to handle childbirth complications; hence, contributing to high rate of infant deaths in Nigeria [32].

Other socioeconomic factors that influence infant deaths were; mothers who had primary or no formal education level, region of residence and ethnicity. Research has documented that education offers mothers increased access to health information and connections with resources for infant health; services, healthcare professionals and effective use of the health care system [33–35]. Education also gives more awareness for good health behaviours. Hence, infants who have educated mothers have lower infant mortality risks. Infants born to mothers from the Northwest region and of Hausa tribe had higher chances of dying has reported in our study. Similar studies from Nigeria reported this finding [25, 36]. Of note is the wide regional disparities in socioeconomic status, health service delivery, resource availability and allocation in Nigeria [36]. The southern states are known to have more health facilities and skilled personnel than the northern states. This region is also characterized with highest proportion of persons with no education which reduces their chances of access to health information [27]. In addition, women in this region start childbearing at younger ages, with possibility of having immature reproductive system; little or no experience with child birth and care. It is also important to note that women in this region have less autonomy compared to women in other regions of the country [27]. Hence, they have no control over household resources which could enhance their health seeking behaviour and health service utilisation.

Our study also found that non-use of contraceptive commodities among the mothers increased the risk of infant mortality as compared with mothers that used contraceptives. Non-use of contraceptives expose women to the risk of unplanned and undesired births which may account for high parity and subject the infants to neglect and improper care especially in the midst of ravaging poverty [37]. This further emphasised the low contraceptive prevalence rate in Nigeria as about 15 % of women are currently using contraceptive [38]. Low contraceptive usage may lead to a quick pregnancy state which often does not allow the mother to have full physical, psychological, physiological and anatomical recovery from previous births. Thus, she may not be fully prepared for subsequent pregnancy and births in terms of her reproductive and socioeconomic capacity. This may have a huge impact on maternal health with resultant birth of infants with health challenges coupled with inadequate care [39].

Aside the sampling errors that can limit the extent to which the findings from this study can be generalized to the entire population, the cross-sectional nature of the data used for this study inhibits the causal relationship between HrBBs and infant mortality. To attribute infant death to high risk birth behavior, further studies that will use cohort or quasi-experimental design approach are thus recommended. The use of a large nationally representative data remains a strength of this study.

## Conclusions

HrBBs is an important predictor of infant mortality and multiple HrBBs increase the chances of death among infants in Nigeria. Screening women for HrBBs for special health attention during pregnancy, birth and postnatal period is essential in alleviating infant death in Nigeria. It is important to create awareness and health education for women, men and families on risk factors of infant deaths such as maternal age, short birth interval and high parity . There is also a need to design interventions to improve female education and economic empowerment which could delay age at marriage for woman till 18 years, when they are matured to take decisions and capable of reproductive health events. At the same time, women will benefit from more awareness and counselling on the risks associated with late childbearing.

## Data Availability

The datasets generated and/or analysed during the current study are available in the [The DHS Program] repository, [http://dhsprogram.com/pubs/pdf/FR359/FR359.pdf].

## Abbreviations

HrBBs: High risk birth behaviours
IMR: Infant mortality rate
NDHS: Nigerian demographic health survey
TFR: Total fertility rate
ANC: Antenatal care
SDG: Sustainable development goal
CPH: Cox Proportional Hazards
